# Measuring mental health and well-being of South African undergraduate students

**DOI:** 10.1017/gmh.2020.26

**Published:** 2020-12-01

**Authors:** Irma Eloff, Marien Graham

**Affiliations:** 1 Department of Educational Psychology, University of Pretoria, Pretoria 0002, South Africa; 2 Department of Science, Mathematics and Technology Education, University of Pretoria, Pretoria 0002, South Africa

**Keywords:** Student mental health, student support, student well-being

## Abstract

**Background:**

Increased investment in optimal student mental health and well-being has been noted by universities around the world. Studies show the need for contextually relevant, granular understandings of specific aspects of student mental health and well-being.

**Methods:**

A survey was conducted at two time points – at the beginning and end of the academic year – at a large, urban university in South Africa. The Mental Health Continuum-Short Form, the Flourishing Scale, and the Fragility of Happiness Scale were used in the testing of undergraduate students from a variety of scientific disciplines. Two separate comparisons were made, based on the baseline data (*n* = 551) and the follow-up data (*n* = 281). In Comparison 1 (baseline, *n* = 443; follow-up, *n* = 173), two independent, biographically (very) similar groups were compared. Comparison 2 (*n* = 108) compared the results from the baseline and follow-up of the same group of students who completed the instruments at both time points.

**Results:**

Results indicate a significant decline in mental health and well-being for both groups (independent and dependent) over the course of the academic year. Both follow-up groups were found to have lower psychological, emotional and social well-being, psychological flourishing, and reduced mental health, in comparison with the baseline groups.

**Conclusions:**

The statistically significant decreases in the mental health and well-being of participants in this study indicate the need for substantive interventions to support student mental health and well-being. Strong foci for well-being interventions should include self-efficacy, sense of direction, meaning and creating a sense of belonging.

## Introduction

Access to higher education has been a key driver for the development of democracies in Africa. In South Africa, universities have been critical partners in addressing societal inequality, overcoming the ravages of apartheid and seeking the well-being of individuals, families and broader communities by providing opportunities for talented youth.

However, the massification of higher education has also increased concerns about student mental health and well-being, as well as about the capacity of universities to provide optimal support to their students. Research has shown that the number of students in need of treatment for mental health disorders far exceeds the resources of most counselling centres and institutions. This leads to a significant unmet need for the treatment of mental health disorders among students (Auerbach *et al*., 2016; Xiao *et al*., 2017). In fact, Auerbach *et al*. (2016), who used 23 World Health Organization (WHO) World Mental Health Surveys carried out in 21 countries to examine mental health disorders of students, concluded that only a small minority of such students receive even minimally adequate treatment. Arguments that an investment in student well-being should at least be on par with investments in the academic future of students (Brooker *et al*., 2019) reiterate the fact that today's university students may well hold influential positions in society in the future. The term ‘well-being’ is well-defined by Dalziel *et al*. (2018, pp. 31–35) in that well-being is understood not simply as positive emotions, but as thriving across multiple domains of life. In this study, the concepts of student mental health and student well-being are positioned to be closely related, even within its distinctiveness. The study adopts the ‘two continua’ model of Westerhof and Keyes (2010, p. 110) in which mental health is not merely viewed as the absence of psychopathologies, but rather in terms of ‘three core components of positive mental health: feelings of happiness and satisfaction with life (emotional well-being), positive individual functioning in terms of self-realisation (psychological well-being), and positive societal functioning in terms of being of social value (social well-being)’.

The mental health and well-being of students (or lack of it) may eventually affect well-being at the systemic level. Current students are the potential employers of the future, who will make strategic decisions within work environments to give priority to the well-being of employees and stakeholders. In addition, students will play critical role players in ensuring sustainable development globally. The latter is articulated in Sustainable Development Goal 3 on Good Health and Well-being, as well as in subsequent progress reports (United Nations, 2019) that are aligned with an emerging international imperative aimed at improving global mental health and well-being (Davidson, 2019; Van Zyl and Rothman, 2019).

The groundswell of studies in this regard has shown the power of positive mental health and well-being to predict long-term outcomes such as quality of life, physical health and longevity, reduced drug and alcohol use, reduced criminality, higher employment, higher lifetime earnings and pro-social behaviour (e.g. volunteering) (Friedli, 2009; Hanlon and Carlisle, 2008; Harward, 2016). Studying the positive mental health and well-being of university students is therefore important within the global context. Several studies indicate that changes in positive mental health, as measured by the Mental Health Continuum-Short Form (MHC-SF), predict the risk of future anxiety and depression disorders. For instance, flourishing (a key indicator of well-being) has been associated with lower prevalence and incidence of depressive and anxiety disorders over 1-year (Grant *et al*., 2013; Lamers *et al*., 2015), 2-year (Keyes *et al*., 2020*b*), 3-year (Schotanus-Dijkstra *et al*., 2017) and 10-year timespans (Keyes *et al*., 2010).

In South Africa, contextual realities dictate a need for tailored assessments of student mental health and well-being on country, regional and institutional levels, since these have been turbulent in the tertiary sector in recent years. Global studies on well-being have consistently shown differences in well-being measures at the country level (Delle Fave *et al*., 2011; Helliwell *et al*., 2020; Keyes *et al*., 2020*a*) and some studies have also indicated differences in individual well-being within regions (Helliwell *et al*., 2018; Walker, 2020). Other studies have adapted well-being measures to be contextually relevant (Wissing *et al*., 2010). It is within this realm that the current study was conducted. Its objective was to assess the mental health and well-being of undergraduate students at a large urban residential university in South Africa over the course of an academic year.

### Hypotheses

*H*_o_: There are no statistically significant differences between the baseline and follow-up groups of undergraduate students in terms of mental health and well-being.

*H*_a_: There are statistically significant differences between the baseline and follow-up groups of undergraduate students in terms of mental health and well-being.

Ethical clearance to conduct the study was obtained from the Ethics Committee of the Faculty of Humanities at the institution involved (GW0180232HS).

## Method

With the objective of this study being to assess the mental health and well-being of undergraduate students in South Africa, a large urban residential university in South Africa was used. This university has just over 100 academic departments offering nearly 1200 study programmes that include a wide range of fields in the humanities, social sciences and natural sciences. The language of instruction is English. The academic year starts in January/February and ends in November/December. Enrolled students have to pay tuition fees and are expected to purchase the necessary prescribed books and equipment for their studies (some also have to pay for room and board). Students do not have to spend any of their funding on internet costs, as this university has free WiFi. This university also supports student well-being by having a variety of programmes focusing on student nutrition, progress, counselling and health. Many studies have shown that increased financial pressure affects students' well-being negatively (Richardson *et al*., 2017; Benson-Egglenton, 2019). University residences are home to students from all undergraduate year groups.

The objective of the study is two-pronged in that it aimed at comparing the mental health and well-being of the same set of undergraduate students (dependent group) between the beginning and the end of one academic year, and at investigating the mental health and well-being for different sets of students (independent group) over time. An attrition analysis was also conducted to investigate whether the students who did not participate at the end of the study had the same level of mental health, the same level of social–psychological prosperity and the same feelings about the fragility of happiness as those who completed the measures at both time points.

### Design

Three instruments, namely the MHC-SF, the Flourishing Scale (FS) and the Fragility of Happiness Scale (FOHS), were combined in electronic format and distributed in English. The MHC-SF is a shorter version of the long-form (MHC-LF) and has been used in the South African context (Keyes *et al*., 2008). The MHC-SF consists of only 14 items, whereas the MHC-LF has 40 items. The MHC-SF comprises three items representing emotional well-being, six representing psychological well-being and five representing social well-being. The instrument measures on a six-point Likert scale, ranging from 1 = ‘never'; 2 = ‘once or twice'; 3 = ‘about once a week'; 4 = ‘about two or three times a week'; 5 = ‘almost every day'; and 6 = ‘every day'. When considering each item on its own, it was found that the higher the value on the Likert scale, the better was the mental health of the respondent for that specific item. Although the individual items on the scale were analysed in this study, an overall score was also computed. This overall score could range from 14 (a respondent selects ‘never’ for all items) to 84 (a respondent selects ‘every day’ for all items). The higher the overall score, the better the overall mental health of the person. The MHC-SF has shown excellent internal consistency and discriminant validity in adolescents (ages 12–18) and adults (18 years and older) in the USA, the Netherlands, and South Africa (Keyes, 2005, 2006; Westerhof & Keyes, 2010; Lamers *et al*., 2011).

The FS (Diener *et al*., 2009) is a measure of psychosocial flourishing designed to measure social–psychological prosperity. Psychological needs such as the need for competence, self-reliance, self-esteem, purpose, optimism, etc., are measured by this scale, which consists of eight items that are measured on a 7-point Likert scale ranging from ‘strongly disagree’ to ‘strongly agree’. When we considered each item on its own, it was found that the higher the value on the Likert scale, the higher was the social–psychological prosperity of the respondent for that specific item. For the FS, Diener *et al*. (2009) suggested that the responses be added for all eight items, which creates an overall score with a minimum value of 8 (a respondent strongly disagrees with all items) and a maximum value of 56 (a respondent strongly agrees with all items). The higher the overall score, the higher was the overall social–psychological prosperity of the person. The FS strongly correlates with other psychological well-being scales (Diener *et al*., 2009). Convergent validity of the scale was established, with strong to weak correlations between the FS and overall well-being, as well as between social and psychological well-being (Schotanus-Dijkstra *et al*., 2016). The same study (Schotanus-Dijkstra *et al*., 2016) though found low correlations in comparison with the MHC-SF.

The FOHS (Joshanloo *et al*., 2015) measures the fragility of happiness beliefs and consists of four items that are measured on a seven-point Likert scale – also ranging from ‘strongly disagree’ to ‘strongly agree’. Regarding each item on its own, we found that the higher the value on the Likert scale, the more strongly the respondent agreed that happiness is fragile or fleeting. The individual items on this scale were also analysed in this study, and an overall score was computed that could range from 4 (a respondent strongly disagrees with all items) to 28 (a respondent strongly agrees with all items). The higher the overall score, the higher was the overall belief that happiness is fragile. The validity, reliability and measurement invariance have been demonstrated widely in at least 15 cultural contexts (Joshanloo *et al*., 2015).

For the present study, Cronbach *α* was used to test the reliability of the instruments, as Cronbach *α* is a measure of internal consistency and values above 0.7 are acceptable (Field, 2018, p. 823). The Cronbach *α* values for the MHC-SF, FS and FOHS were equal to 0.930, 0.876 and 0.824, respectively, indicating that the short form and the two scales are reliable.

### Study setting and data collection

The instruments were distributed electronically to registered undergraduate students who were staying in university residences at the time of the study. Student leaders (who were working in student well-being portfolios) invited students to participate in the study at both baseline and follow-up. The same recruitment strategy was used at baseline and follow-up. Therefore the same group of students who were invited to participate at baseline were again invited to participate at follow-up. Altogether 551 undergraduate students participated in the baseline study, while 281 students from the same university participated in the follow-up study. The lower response rate at follow-up was attributed to the pending examination period. Participation was widely encouraged by student leaders in the residences at both time points. The study was conducted between February 2019 (baseline) and September/October 2019 (follow-up).

### Data analysis

Data were analysed using the Statistical Package for the Social Sciences (SPSS), Version 26. In the case of individual Likert scale items and for the overall scores, two comparative data analysis processes were conducted using the Mann–Whitney (MW) test and the Wilcoxon signed-rank (WSR) test for the independent (unrelated) and dependent (related) group comparisons, respectively. Since the overall scores are continuous variables, the Shapiro–Wilk test was run to test for normality. This test showed that the overall scores were not normally distributed (*p* < 0.05) and, accordingly, non-parametric tests were used to compare the results of the unrelated (the MW test) and the related groups (the WSR test), respectively. For the nominal categorical biographical variables, the χ^2^ test was used to conduct comparative data analysis. The first comparison was between two unrelated groups of students who presented no significant differences with regard to their nominal categorical biographical variables (province, citizenship, gender, race and home language). In Comparison 1, the group which completed the instruments at baseline (*n* = 443) was unrelated to the group who completed the instruments at follow-up (*n* = 173), but the two groups were very similar in terms of their biographic variables. In Comparison 2 (the related group comparison), results from a group of students (*n* = 108) who completed the instruments at both baseline and follow-up were analysed.

## Results

### Biographical variables of the participants

The decision on the inclusion of specific demographic variables for the study was derived from in-depth consultations with the university executive, senior leaders (deans and deputy deans) and internal stakeholders involved in student support. For the continuous biographical variable, age, normality was tested for using the Shapiro–Wilk test. Since age was not normally distributed (*p* < 0.05), non-parametric tests were used to compare the ages between the baseline and follow-up groups. For Comparison 1, the mean age for the baseline group was 18.79 (median = 18.00, s.d. = 3.133) and the mean age for the follow-up group was 19.20 (median = 19.00, s.d. = 1.175). The MW test indicated that the mean and median ages of the follow-up group were statistically significantly higher than those of the baseline group (MW = 26 295.500, *p* < 0.001). For Comparison 2, the mean age for the baseline group was 18.42 (median = 18.00, s.d. = 0.866) and the mean age for the follow-up group was 19.09 (median = 19.00, s.d. = 1.586).

The WSR test indicated that the mean and median ages of the follow-up group were statistically significantly higher than those of the baseline group (WSR = −7.178, *p* < 0.001). This was expected, as this was the same group of respondents who completed the same questionnaire a substantial amount of time later. The categorical biographical variables in the questionnaire consisted of asking respondents the degree programme for which they were currently enrolled, their province, citizenship, gender, race and home language. All categorical biographical variables, except for degree enrolment (due to the multiplicity of answers), are reported in Table 1. The degrees included a wide variety of baccalaureate and undergraduate programmes. Table 1 summarises the categorical biographical variables of the independent groups from Comparison 1. In Comparison 2, the same group of students who had answered the questionnaires at both baseline and follow-up was used. Therefore, only the frequencies of the biographical data are provided for the 108 responses.

**Table 1.** Cross-tabulation of biographical variables for unrelated and related groups of students

		Comparison 1 (two unrelated groups)			Comparison 2 (Same group)	
	443 (100.0%)	173 (100.0%)	616 (100.0%)	108	100%
Total *n* (% within group)	Baseline	Follow-up	Total	Frequency	Percentage
*Province*						
Eastern Cape	*n* (% within group)	11 (2.5%)	2 (1.2%)	13 (2.1%)	5	4.6
Free State	*n* (% within group)	12 (2.7%)	7 (4.0%)	19 (3.1%)	3	2.5
Gauteng	*n* (% within group)	224 (50.6%)	83 (48.0%)	307 (49.8%)	54	50.0
KwaZulu-Natal	*n* (% within group)	67 (15.1%)	20 (11.6%)	87 (14.1%)	15	13.9
Limpopo	*n* (% within group)	46 (10.4%)	20 (11.6%)	66 (10.7%)	6	5.6
Mpumulanga	*n* (% within group)	47 (10.6%)	21 (12.1%)	68 (11.0%)	21	19.4
North West	*n* (% within group)	26 (5.9%)	12 (6.9%)	38 (6.2%)	1	0.9
Western Cape	*n* (% within group)	10 (2.3%)	8 (4.6%)	18 (2.9%)	3	2.8
*Citizenship*						
SA citizen	*n* (% within group)	419 (94.6%)	168 (97.1%)	587 (95.3%)	107	99.1
SADC country	*n* (% within group)	12 (2.7%)	4 (2.3%)	16 (2.6%)	1	0.9
Non-African student	*n* (% within group)	8 (1.8%)	1 (0.6%)	9 (1.5%)		
Other African countries	*n* (% within group)	4 (0.9%)	0 (0.0%)	4 (0.6%)		
*Gender*						
Male	*n* (% within group)	159 (35.9%)^a^	42(24.3%)^a^	201 (32.6%)	56	51.9
Female	*n* (% within group)	282 (63.7%)^a^	129 (74.6%)^a^	411 (66.7%)	52	48.1
Missing	*n* (% within group)	2 (0.5%)	2 (1.2%)	4 (0.6%)	108	100
*Race*						
African	*n* (% within group)	159 (35.9%)	68 (39.3%)	227 (36.9%)	29	26.9
Coloured^b^	*n* (% within group)	18 (4.1%)	7 (4.0%)	25 (4.1%)	3	2.8
Indian	*n* (% within group)	16 (3.6%)	8 (4.6%)	24 (3.9%)	8	7.4
White	*n* (% within group)	240 (54.2%)	88 (50.9%)	328 (53.2%)	66	61.1
Others	*n* (% within group)	10 (2.3%)	2 (1.2%)	12 (1.9%)	2	1.9
*Home language*						
Afrikaans	*n* (% within group)	135 (30.5%)	61 (35.3%)	196 (31.8%)	36	33.3
English	*n* (% within group)	154 (34.8%)	51 (29.5%)	205 (33.3%)	46	42.6
IsiZulu (Zulu)	*n* (% within group)	26 (5.9%)	17 (9.8%)	43 (7.0%)	4	3.7
Northern Sotho (Sepedi)	*n* (% within group)	32 (7.2%)	16 (9.2%)	48 (7.8%)	3	2.8
Setswana (Tswana)	*n* (% within group)	25 (5.6%)	6 (3.5%)	31 (5.0%)	2	1.9
Tshivenda (Venda)	*n* (% within group)	4 (0.9%)^a^	6 (3.5%)^a^	10 (1.6%)	1	0.9
Sesotho (Southern Sotho)	*n* (% within group)	15 (3.4%)	2 (1.2%)	17 (2.8%)	3	2.8
SiSwati (Swati)	*n* (% within group)	9 (2.0%)	1 (0.6%)	10 (1.6%)	5	4.6
IsiNdebele (Ndebele)	*n* (% within group)	3 (0.7%)	1 (0.6%)	4 (0.6%)	3	2.8
Xitsonga (Tsonga)	*n* (% within group)	4 (0.9%)	5 (2.9%)	9 (1.5%)	1	0.9
IsiXhosa (Xhosa)	*n* (% within group)	17 (3.8%)	3 (1.7%)	20 (3.2%)	3	2.8
Others	*n* (% within group)	19 (4.3%)	4 (2.3%)	23 (3.7%)	1	0.9

a The column proportions differ statistically significantly from one another.

b The term ‘coloured’ is a highly contested term. The term is sometimes used to describe a person of mixed European (‘White’) and African (‘Black’) or Asian ancestry (Encyclopaedia Britannica, 1998). In this study, it was used in the biographical variable section of data collection, in alignment with the categories utilised by Statistics South Africa (http://www.statssa.gov.za/).

For Comparison 1, since all the *p* values were >0.05 for province (χ^2^ = 6.085, *p* = 0.530), citizenship (χ^2^ = 3.005, *p* = 0.391) and race (χ^2^ = 1.752, *p* = 0.781), there were no statistically significant differences between the biographical data (province, citizenship and race) of the two groups. However, for home language (χ^2^ = 21.839, *p* = 0.026) there was a statistically significant difference between the proportion of Tshivenda (Venda) speaking students with the follow-up group having significantly more Tshivenda (Venda) speaking students. Note, however, out of 11 languages that were considered in this study, only the proportion of Tshivenda (Venda) speaking students differed significantly between the baseline and follow-up groups.

Gender also showed a statistically significant difference (χ^2^ = 7.379, *p* = 0.007) between the groups with the follow-up group having significantly more females than the baseline group. Table 1 indicates that the gender distribution of the respondents was almost 50/50 for this survey, which is representative of the total student population at this university (its population gender distribution was also ~50/50). In terms of race, it should be noted that the majority of respondents in the survey were white, whereas the population student profile for this university indicated that the majority of students are black. This implies that, in terms of race, the sample is not representative of the population study body. The total undergraduate student population during the year of data collection (2019) consisted of African (48.7%), coloured (3.0%), Indian (6.4%) and white (41.9%). (The study acknowledges race as a social construction; however, in the historical context of South Africa, it was deemed important to capture students' racial self-identification in the biographical section, in alignment with Statistics South Africa, 2020).

### Findings on student mental health and well-being

For the Likert-type questions in the MHC-SF, FS and FOHS, the MW test was used to compare the responses of the two independent groups in Comparison 1. In Comparison 2, the WSR test was used to compare the responses when the same group completed the questionnaires (i.e. MHC-SF, FS and FOHS) at different time points during the academic year.

To start with, the individual items, the overall score, the descriptive statistics, and the results of the MW and the WSR tests for the MHC-SF for both comparisons are summarised in Table 2.

**Table 2.** Results of the MHC-SF for related and unrelated groups

Item	Comparison 1 (two unrelated groups at baseline and follow-up)			Comparison 2 (same group at baseline and follow-up)		
During the past month, how often did you feel:	Baseline	Follow-up	MW; *p* value	Baseline	Follow-up	WSR; *p* value
	Mean; median (S.D.)	Mean; median (S.D.)		Mean; median (S.D.)	Mean; median (S.D.)	
Happy?	4.52; 5.00 (1.175)	4.34; 5.00 (1.143)	33 851.000; 0.016*	4.70; 5.00 (1.044)	4.48; 5.00 (1.072)	−2.047; 0.041*
Interested in life?	4.67; 5.00 (1.284)	4.38; 5.00 (1.255)	32 390.500; 0.002*	4.85; 5.00 (1.183)	4.54; 5.00 (1.314)	−2.782; 0.005*
Satisfied with life?	4.37; 5.00 (1.327)	4.06; 4.00 (1.455)	33 802.500; 0.017*	4.37; 5.00 (1.418)	4.25; 5.00 (1.395)	−1.183; 0.237
That you had something important to contribute to society?	3.92; 4.00 (1.424)	3.76; 4.00 (1.532)	36 118.000; 0.255	4.05; 4.00 (1.506)	3.85; 4.00 (1.521)	−1.805; 0.071
That you belonged to a community?	4.39; 5.00 (1.507)	4.03; 5.00 (1.566)	33 040.500; 0.006*	4.66; 5.00 (1.382)	4.22; 5.00 (1.579)	−3.411; 0.001*
That our society is a good place or is becoming a better place for all people?	3.61; 4.00 (1.459)	2.70; 2.00 (1.582)	25 684.500; 0.000*	3.71; 4.00 (1.559)	3.13; 3.00 (1.624)	−3.517; 0.000*
That people are basically good?	4.04; 4.00 (1.248)	3.31; 3.00 (1.349)	26 360.500; 0.000*	4.21; 5.00 (1.408)	3.72; 4.00 (1.446)	−3.231; 0.001*
That the way our society works makes sense to you?	3.48; 4.00 (1.445)	2.64; 2.00 (1.544)	26 293.000; 0.000*	3.31; 4.00 (1.626)	2.95; 3.00 (1.597)	−1.712; 0.087
That you liked most parts of your personality?	4.49; 5.00 (1.310)	4.27; 5.00 (1.333)	34 195.000; 0.031*	4.39; 5.00 (1.426)	4.25; 5.00 (1.441)	−1.287; 0.198
Good at managing the responsibilities of your daily life?	4.04; 4.00 (1.319)	3.72; 4.00 (1.429)	33 294.000; 0.009*	3.94; 4.00 (1.459)	3.92; 4.00 (1.422)	−0.093; 0.926
That you had warm and trusting relationships with others?	4.54; 5.00 (1.316)	4.32; 5.00 (1.410)	34 893.500; 0.072	4.46; 5.00 (1.363)	4.44; 5.00 (1.313)	−0.131; 0.896
That you had experiences that challenged you to grow and become a better person?	4.56; 5.00 (1.225)	4.25; 5.00 (1.374)	33 937.500; 0.002*	4.68; 5.00 (1.303)	4.56; 5.00 (1.320)	−0.748; 0.455
Confident to think and express your own ideas and opinions?	4.27; 5.00 (1.366)	4.02;4.00 (1.445)	34 716.000; 0.062	4.19; 4.00 (1.382)	4.15; 5.00 (1.509)	−0.273; 0.785
That your life has a sense of direction or meaning to it?	4.49; 5.00 (1.397)	4.17; 4.00 (1.529)	33 810.000; 0.019*	4.53; 5.00 (1.397)	4.28; 5.00 (1.497)	−1.946; 0.052
Overall score	59.38; 62.00 (13.722)	53.97; 56.00 (14.145)	28 809.500; 0.000*	60.05; 64.00 (14.458)	56.74; 58.00 (16.066)	−2.945; 0.003*

*For *p* values <0.05, the null hypothesis was rejected and statistically significant differences were found.

From Table 2, it is clear that statistically significant differences occurred between the responses of the baseline and follow-up groups for 11 of the 14 items in Comparison 1 (i.e. unrelated group). For the 11 items, the baseline responses indicated better mental health for these participants than did the follow-up responses. The bottom row of Table 2 shows the results of the overall score, and for Comparison 1 it can be seen that the mean (and median) of the responses of the baseline group were statistically significantly higher than those of the follow-up group. This indicates better mental health for the baseline responses (unrelated group). In Comparison 2, statistically significant differences were found between the responses of the baseline and follow-up groups for five of the items on the MHC-SF (related group). In Comparison 2, the bottom row of Table 2 shows that the mean (and median) of the responses of the baseline group were statistically significantly higher than those of the follow-up group, which indicated better mental health for the baseline responses (related group). Thus, for the MHC-SF, the baseline responses indicated better mental health and well-being, based on the follow-up responses in both comparisons.

An item analysis of the *two independent groups* on the MHC-SF shows that from the beginning to the end of the academic year, students' feelings of well-being, as well as their interests in and satisfaction with life declined significantly. They also felt less part of a community than before and felt that ‘our society is a good place or is becoming a better place for all people’ to a lesser extent than earlier in the academic year. Furthermore, they felt that ‘people are basically good’ statistically less so and also that ‘the way our society works makes sense to you’ to a lesser extent than at the start of the academic year. Other items that differed statistically significantly included the following: students felt less that they liked most parts of their personality; they felt less that they were good at managing the responsibilities of their daily lives; they felt less that they had experiences that challenged them to grow and become a better person, and they felt less that their lives had a sense of direction and meaning to it.

Similarly, an item analysis of the *same group of students* on the MHC-SF shows that significant differences emerged from the beginning to the end of the academic year in that students felt less happy, less interested in life and less part of a community. As with the two independent groups, this group of students also felt to a lesser extent that ‘our society is a good place or is becoming a better place for all people’ and that ‘people are basically good’ than they did earlier in the year.

Next, the individual items, the overall score, the descriptive statistics, and the results of the MW and the WSR tests for the FS for both comparisons are summarised in Table 3.

**Table 3.** Results of the FS for unrelated and related groups of students

Item	Comparison 1 (two unrelated groups at baseline and follow-up)			Comparison 2 (same group at baseline and follow-up)		
	Baseline	Follow-up	MW; *p* value	Baseline	Follow-up	WSR; *p* value
	Mean; median (S.D.)	Mean; median (S.D.)		Mean; median (S.D.)	Mean; median (S.D.)	
I lead a purposeful and meaningful life	6.00; 6.00 (1.046)	5.77; 6.00 (1.321)	35 661.000; 0.150	6.11; 6.00 (0.921)	5.75; 6.00 (1.422)	−2.395; 0.017*
My social relationships are supportive and rewarding	5.95; 6.00 (1.044)	5.87; 6.00 (1.144)	36 970.000; 0.467	5.87; 6.00 (1.111)	5.92; 6.00 (1.015)	−0.618; 0.537
I am engaged and interested in my daily activities	5.72; 6.00 (1.154)	5.41; 6.00 (1.376)	33 693.000; 0.013*	5.84; 6.00 (1.129)	5.41; 6.00 (1.473)	−3.061; 0.002*
I actively contribute to the happiness and well-being of others	5.92; 6.00 (0.963)	5.88; 6.00 (1.0470)	37 951.500; 0.841	6.02; 6.00 (0.907)	6.04; 6.00 (1.041)	−0.402; 0.688
I am competent and capable in the activities that are important to me	6.12; 6.00 (0.955)	5.87; 6.00 (1.161)	33 601.000; 0.010*	6.01; 6.00 (0.837)	5.77; 6.00 (1.173)	−2.144; 0.032*
I am a good person and live a good life	6.03; 6.00 (0.961)	5.77; 6.00 (1.201)	33 858.500; 0.015*	6.09; 6.00 (0.981)	5.72; 6.00 (1.237)	−3.142; 0.002*
I am optimistic about my future	6.07; 6.00 (1.067)	5.87; 6.00 (1.306)	35 714.000; 0.162	6.09; 6.00 (1.124)	5.67; 6.00 (1.421)	−2.973; 0.003*
People respect me	5.69; 6.00 (1.007)	5.66; 6.00 (1.097)	38 182.000; 0.940	5.68; 6.00 (1.167)	5.57; 6.00 (1.146)	−0.700; 0.484
Overall score	47.51; 48.00 (5.865)	46.11; 48.00 (7.378)	34 352.000; 0.045*	42.04; 43.00 (5.283)	40.27; 42.00 (7.101)	−2.844; 0.004*

*For *p* values <0.05, the null hypothesis was rejected and statistically significant differences were found.

In Comparison 1, statistically significant differences were found between the responses of the baseline and follow-up groups for three of the eight items on the FS. The bottom row of Table 3 shows that the overall score of Comparison 1 differed significantly between the baseline and follow-up groups, with the mean (and median) of the baseline group being significantly higher. This implied higher social–psychological prosperity for the baseline responses (unrelated group).

In Comparison 2, statistically significant differences emerged between the responses of the baseline and follow-up groups for five of the eight items. The bottom row of Table 3 shows that for Comparison 2, the mean (and median) of the responses of the baseline group were statistically significantly higher than those of the follow-up group, which indicates higher social–psychological prosperity for the baseline responses (related group). For each time point, the baseline responses indicated that students were flourishing more at baseline than during the follow-up in both comparisons. When considering the results of the comparisons between the unrelated and the related group for the FS, the baseline group displayed significantly higher social–psychological prosperity than the follow-up group for both comparisons.

An item analysis shows that students in the independent groups were significantly less interested in daily life and activities; they felt less capable and competent in activities that were important to them to a lesser degree than earlier in the year. They reacted similarly negatively to the statement, ‘I am a good person and live a good life’. The item analysis for the same group of students shows similar responses to these three items. In addition, declines can also be detected on the items that measure whether students feel that they ‘lead a purposeful and meaningful life’ and ‘I am optimistic about my future’.

To summarise, the individual items, the overall score, the descriptive statistics, and the results of the MW and WSR tests for the FOHS for both comparisons are listed in Table 4.

**Table 4.** Results of the FOHS for unrelated and related groups

Item	Comparison 1 (two unrelated groups at baseline and follow-up)			Comparison 2 (same group at baseline and follow-up)		
	Baseline	Follow-up	MW; *p* value	Baseline	Follow-up	WSR; *p* value
	Mean; median (S.D.)	Mean; median (S.D.)		Mean; median (S.D.)	Mean; median (S.D.)	
Something might happen at any time and we could easily lose our happiness	5.29; 5.00 (1.512)	5.34; 6.00 (1.575)	37 153.000; 0.547	5.27; 6.00 (1.425)	5.22; 6.00 (1.561)	−0.115; 0.909
Happiness is fragile	4.96; 5.00 (1.617)	4.91; 5.00 (1.680)	38 048.000; 0.889	4.96; 5.00 (1.734)	4.94; 6.00 (1.758)	−0.191; 0.849
It is likely that our happiness could be reduced to unhappiness with a simple accident	5.28; 6.00 (1.509)	5.29; 6.00 (1.621)	37 056.000; 0.512	4.98; 5.00 (1.702)	5.22; 6.00 (1.625)	−2.012; 0.044*
There is only a thin line between happiness and unhappiness	4.35; 5.00 (1.842)	4.30; 5.00 (1.831)	37 709.500; 0.756	4.12; 4.00 (1.947)	4.34; 4.00 (1.915)	−1.410; 0.158
Overall score	19.88; 20.00 (5.224)	19.84; 21.00 (5.521)	37 858.000; 0.816	19.33; 20.00 (5.732)	19.72; 21.00 (6.076)	−1.244; 0.213

*For *p* values <0.05, the null hypothesis was rejected and statistically significant differences were found.

Table 4 shows that for the FOHS, no significant differences in responses were found between any of the individual items or between the overall scores of the baseline and follow-up groups in Comparison 1. In Comparison 2, there were also no significant differences between the overall score or any of the individual items, except for one item: the follow-up group agreed significantly stronger with the statement that ‘happiness could be reduced to unhappiness with a simple accident’. This being said, it is worthy to note that responses for all the other items on the FOHS did not differ significantly between the baseline and follow-up groups. After all, the belief that happiness is fragile is common across individuals and cultures.

It was furthermore interesting to note that the FOHS was the only scale where the responses between the baseline and follow-up groups remained very similar. No statistically significant differences were found for the independent (unrelated) group, while for the dependent (related) group, a decrease was found for only one item, namely ‘It is likely that our happiness could be reduced to unhappiness with a simple accident’. Therefore, the view that happiness is temporary (or fleeting) and can easily shift to a neutral or less favourable state, remained relatively unchanged at both time points for both groups. In terms of the undergraduate student experience, our study suggests that this commonality may provide a springboard for planning well-being interventions.

Finally, an attrition analysis was conducted to investigate whether the students who did not participate at the end of the study had the same level of mental health, the same level of social–psychological prosperity and the same feelings about the fragility of happiness as those who completed the measures at both time points; this was done by comparing the overall MHC-SF baseline scores, the overall FS baseline scores and the overall FOHS baseline scores, respectively, between these two groups of students. The MW tests showed no statistically significant differences between the overall mean scores for the MHC-SF (MW =  23 026.500, *p* = 0.546) and the FOHS (MW =  22 877.000, *p* = 0.481), respectively. However, when comparing the overall FS baseline scores, the MW test showed a statistically significant difference (MW = 9336.000, *p* < 0.001) between the overall mean scores of 47.51 (median = 48.00, s.d. = 5.865) for students who completed the questionnaire only once and 42.04 (median = 43.00, s.d. = 5.283) for students who completed the questionnaire at both time points. This shows that the students who completed the questionnaire at both time points had a lower level of social–psychological prosperity than those who completed the questionnaire only once.

Table 5 shows the correlations between the overall scores. As expected, there are statistically significant positive correlations between the MHC-SF and FS overall scores, since the higher the overall score for MHC-SF, the better was the students' mental health. Also, the higher the overall score for FS, the higher was the social–psychological prosperity. This correlation is in line with the view that a person who has good mental health will also experience psychological prosperity. Similarly, the FOHS overall score correlates statistically significantly negatively with the other scores, because the higher the overall score for FOHS, the stronger was the students' belief that happiness is fragile.

**Table 5.** Spearman correlations between overall scores

Correlations between	Baseline		Follow-up	
	Correlation	*p* value	Correlation	*p* value
Comparison 1 (two independent groups at baseline and follow-up)				
MHC-SF and FS	0.584	0.000*	0.679	0.000*
MHC-SF and FOHS	−0.347	0.000*	−0.411	0.000*
FS and FOHS	−0.181	0.000*	−0.420	0.000*
Comparison 2 (same group at baseline and follow-up)				
MHC-SF and FS	0.632	0.000*	0.639	0.000*
MHC-SF and FOHS	−0.408	0.000*	−0.520	0.000*
FS and FOHS	−0.266	0.005*	−0.475	0.000*

* For *p* values <0.05, statistically significant correlations were found.

Research has shown that female students tend to experience more mental health challenges than male students (Auerbach *et al*., 2018; Bantjes *et al*., 2019; Gitay *et al*., 2019; Van der Walt *et al*., 2020). In the present study, gender differences were investigated for the overall scores. For the sake of conciseness, the individual item analysis is not included in this report. In the case of Comparison 1 (unrelated group), there were no significant differences between gender for the overall FS and the overall FOHS scores, or the baseline and follow-up data. However, for the MHC-SF, statistically significant differences were found for both the baseline data (MW = 19 018.000, *p* = 0.008) and the follow-up data (MW = 2025.500, *p* = 0.014). Closer scrutiny revealed that males indicated better mental health than females. For the baseline data, the mean of the overall score for the MHC-SF equalled 61.55 (median = 64.00, s.d. = 13.254) for males and 58.24 (median = 61.00, s.d. = 13.738) for females. For the follow-up data, the mean of the overall score for the MHC-SF equalled 58.95 (median = 60.50, s.d. = 12.943) for males and 52.37 (median = 56.00, s.d. = 14.232) for females. In the case of Comparison 2 (related group), we found no statistically significant differences between gender for any of the overall scores.

## Discussion

Results from our study indicated statistically significant decreases in the mental health and well-being indicators of undergraduate students between the beginning and the end of the academic year. These decreases were evident in indicators for independent as well as dependent (same) groups of students across academic disciplines. Overall, both of the follow-up groups in this study experienced lower social–psychological prosperity, as well as decreased mental health and well-being than the baseline groups. Results from the study are in line with studies on students from specific disciplines in other contexts. For instance, a study among medical students in the United States (Ludwig *et al*., 2015) demonstrated a significant increase in the proportion of students who were at risk for depression in their third year, as compared to their first year. Ludwig *et al*. (2015) also measured an increase in perceived stress in these students. A study among students of veterinary sciences in the United Kingdom (Cardwell *et al*., 2013) found that student well-being was significantly poorer (*p* < 0.001) and the degree of mental distress significantly higher (*p* < 0.001) than in general population estimates. Suicidal ideation was also more likely among veterinary science students than among the general population.

The WHO World Mental Health International College Student project is conducting a series of surveys in 19 colleges across eight countries (of which South Africa is one). As part of the project, Auerbach *et al*. (2018) found that roughly one-third of first-year students screened positive for at least one common DSM-IV anxiety, mood, or substance disorder. In a survey conducted among South African undergraduate and postgraduate students, 11.2 and 15.8%, respectively, indicated that they experienced moderate to severe symptoms of depression and anxiety (Bantjes *et al*., 2016).

Based on the above figures, it is evident that the decline in the mental health and well-being of undergraduate students is serious. In-depth item analysis might provide insights into the domain-specific areas of well-being most affected, but the general trend towards decreased well-being calls for wider recognition of the urgency to attend to the mental health and well-being of university students. Worldwide, more than 650 million people are challenged by mental health disorders, with the majority of this burdened group (up to 75%) residing in low- and middle-income countries (Davidson, 2019). Apart from individual benefits, attending to the mental health and well-being of university students in this broader population potentially extends and supports well-being at the societal level. Several researchers (Giroux, 2016; Keyes, 2016; Ryff, 2016; Auerbach *et al*., 2018) have suggested the importance of investigating and supporting the mental health and well-being of university students. Substantive investments had been made in student well-being at the institution where this study was conducted. Our research suggests that the broad downward trend in the mental health and well-being of undergraduate students should be stemmed by focusing on specific indicators of well-being that need support. It also suggests that conversations about student well-being should include their own role in increasing their well-being, to broaden the implicit continuum of support available to students. In this regard, a study on the experiences of people with high/flourishing *v.* low/languishing levels of positive mental health in various geographic locations in South Africa (using the MHC-SF) suggests that ‘languishing people manifested a self-focus and often motivated responses in terms of own needs and hedonic values such as own happiness, whereas flourishers were more other-focused and motivated responses in terms of eudaemonic values focusing on a greater good’ (Wissing *et al.*, 2019:1). In relation to the findings from this study, this notion of moving from an ‘inner’ towards an ‘outer’ focus might potentially be useful in supporting student well-being.

The reasons for the decreases in student mental health and well-being from this study nevertheless warrant serious contemplation. Why are the mental health and well-being of these students declining? In our study, the results could well be related to the proximity of examinations at the end of the year, or due to the mere nature of ‘transition status’ in the lifespan of undergraduate students. Undergraduate studies are a period of identity formation in which key life decisions are made. It is also often a time of intense academic pressure and a pathway towards independence, during which new relationships are formed. These hypotheses all need further empirical investigation. However, holistic approaches towards student mental health and well-being support the notion that student mental health and well-being should ideally improve over time. Individual agency in supporting personal well-being is as important as any support provided at the institutional level.

This study focuses on student well-being rather than mental ill health and provides a specific item analysis for this specific context to plan future well-being interventions. Although the study is situated at one institution, other studies suggest that more fine-grained understandings of student well-being may potentially have long-term benefits.

## Limitations of the study

Our study was limited by its relatively small sample size and consequent poor generalisability, and the lack of qualitative data to elucidate the quantitative data. Since the data was collected during a period that followed shortly after significant turbulence in the university sector in the country, follow-up data with subsequent year groups might be helpful. This period of turbulence – student protests, temporary campus closures at all public universities in South Africa, learning losses, safety concerns, comprehensive financial impacts on the tertiary sector – prior to data collection in this study, could have affected the results from this study. At the same time, results from this study provide a potentially valuable barometer for student well-being following a period of turbulence. Other limitations include the fact that mental health being, social–psychological prosperity, and perceptions on the fragility of happiness can be different for different courses and different faculties, as these exert different pressures on students. For example, some courses are more expensive than others and, as discussed earlier, financial worries may lead to poor mental well-being and low social–psychological prosperity. The fact that the follow-up data were collected during the period leading into the final examinations two months later, may also have affected the data.

## Conclusion

The current study suggests that the mental health and well-being of undergraduate students at university may decrease from the beginning to the end of the year. It also provides insight into the specific aspects of the psychological and social well-being of undergraduate students that may need additional support. Furthermore, the study suggests that individual agency for well-being should be on par with institutional support for student well-being. Our findings might help universities to inform planning processes following the Covid-19 pandemic and the resultant recalibration of tertiary institutions to optimally serve their students.
